# Monitoring Freshman College Experience Through Content Analysis of Tweets: Observational Study

**DOI:** 10.2196/publichealth.7444

**Published:** 2018-01-11

**Authors:** Sam Liu, Miaoqi Zhu, Sean D Young

**Affiliations:** ^1^ School of Exercise Science, Physical and Health Education University of Victoria Victoria, BC Canada; ^2^ College of Computing and Digital Media DePaul University Chicago, IL United States; ^3^ Family Medicine University of California Los Angeles Los Angeles, CA United States

**Keywords:** social networking, big data, population surveillance, education, students, social media, Twitter

## Abstract

**Background:**

Freshman experiences can greatly influence students’ success. Traditional methods of monitoring the freshman experience, such as conducting surveys, can be resource intensive and time consuming. Social media, such as Twitter, enable users to share their daily experiences. Thus, it may be possible to use Twitter to monitor students’ postsecondary experience.

**Objective:**

Our objectives were to (1) describe the proportion of content posted on Twitter by college students relating to academic studies, personal health, and social life throughout the semester; and (2) examine whether the proportion of content differed by demographics and during nonexam versus exam periods.

**Methods:**

Between October 5 and December 11, 2015, we collected tweets from 170 freshmen attending the University of California Los Angeles, California, USA, aged 18 to 20 years. We categorized the tweets into topics related to academic, personal health, and social life using keyword searches. Mann-Whitney *U* and Kruskal-Wallis *H* tests examined whether the content posted differed by sex, ethnicity, and major. The Friedman test determined whether the total number of tweets and percentage of tweets related to academic studies, personal health, and social life differed between nonexam (weeks 1-8) and final exam (weeks 9 and 10) periods.

**Results:**

Participants posted 24,421 tweets during the fall semester. Academic-related tweets (n=3433, 14.06%) were the most prevalent during the entire semester, compared with tweets related to personal health (n=2483, 10.17%) and social life (n=1646, 6.74%). The proportion of academic-related tweets increased during final-exam compared with nonexam periods (mean rank 68.9, mean 18%, standard error (SE) 0.1% vs mean rank 80.7, mean 21%, SE 0.2%; Z=–2.1, *P*=.04). Meanwhile, the proportion of tweets related to social life decreased during final exams compared with nonexam periods (mean rank 70.2, mean 5.4%, SE 0.01% vs mean rank 81.8, mean 7.4%, SE 0.01%; Z=–4.8, *P*<.001). Women tweeted more often than men during both nonexam (mean rank 95.8 vs 76.8; *U*=2876, *P*=.02) and final-exam periods (mean rank 96.2 vs 76.2; *U*=2832, *P*=.01). The percentages of academic-related tweets were similar between ethnic groups during nonexam periods (*P*>.05). However, during the final-exam periods, the percentage of academic tweets was significantly lower among African Americans than whites (χ^2^_4_=15.1, *P*=.004). The percentages of tweets related to academic studies, personal health, and social life were not significantly different between areas of study during nonexam and exam periods (*P*>.05).

**Conclusions:**

The results suggest that the number of tweets related to academic studies and social life fluctuates to reflect real-time events. Student’s ethnicity influenced the proportion of academic-related tweets posted. The findings from this study provide valuable information on the types of information that could be extracted from social media data. This information can be valuable for school administrators and researchers to improve students’ university experience.

## Introduction

The first year of university is a critical period for students to get acclimated to the postsecondary environment [1]. Studies have shown that students’ success is largely influenced by their freshman experiences (eg, academic studies, personal health, and social life) during their first year [1,2]. However, traditional methods of monitoring the freshman experience, such as conducting surveys, can be resource intensive and time consuming. Reports of students’ experiences may not be available until the following semester [3]. Consequently, this can significantly limit the ability of schools to understand students’ experiences in real time and rapidly offer assistance to help improve the students’ experiences.

Social media technology may be well suited to address this challenge. Use of social media, such as Twitter, has been rapidly growing among postsecondary students [4]. In the past 3 years, Twitter use among college students in the United States has increased by 20% [4]. Users often share their daily lives on these social media platforms and, as a result, social media data may be used to provide useful information about student experiences. However, to our knowledge, no studies have examined how frequently postsecondary students talk about topics related to academic studies, personal health, and social life on Twitter. Furthermore, little is known about whether these topic discussions differ by demographics (eg, sex, ethnicity, and major area of study) or whether the topic discussion changes throughout the semester (eg, nonexam vs exam periods). Understanding the types of content and frequency of content discussion is an important first step to determining whether it is feasible to use Twitter data to monitor students’ college experience in real time. Therefore, the primary objective of this study was to describe the proportion of content posted on Twitter by college students relating to academic studies, personal health, and social life throughout the semester. The secondary objective was to examine whether the proportion of content differed by demographics and during nonexam versus exam periods.

## Methods

### Overview

This was an observational study that took place from September 21, 2015 to December 11, 2015. We recruited 197 first-year undergraduate freshman students at the University of California Los Angeles (UCLA), California, USA, aged 18 to 20 years. A total of 170 participants were active Twitter users who posted at least 3 tweets per week, and they were included in the analysis. We obtained ethics approval from the UCLA Research Ethics Board.

### Recruitment and Study Protocol

We recruited participants from September 21 to October 4, 2015. Participants were informed about the study through flyers on social media websites and on the UCLA campus. Eligible participants who provided consent were asked to complete an online questionnaire that assessed their demographic characteristics, which included age, sex, ethnicity, and area of study. Participants were asked to share their Twitter handle. We extracted all participants’ tweets from October 5 to December 11, 2015, for analysis using the Twitter streaming application program interface.

### Content Analysis

We converted data to lowercase and removed punctuation. We then stemmed all words to remove suffixes; for example, “studied” and “studying” simply became “study.” All English stop words were also removed from the data. Any retweeted tweets using the notation “RT” were excluded to prevent popular posts or spam from saturating the sample. Only English tweets were included in our analyses. We then created an algorithm that counted the most frequently used words. Using an iterative process, we manually categorized the words into topic contents related to academic, personal health, and social life. Each tweet was then classified, using a search algorithm, into the topics if it contained at least one keyword. The topics were nonmutually exclusive. A sample of randomly selected (20%) tweets was manually checked to ensure they were accurately related to the categories.

### Statistical Analysis

We used descriptive statistics to tabulate types of tweets. Our data were not normally distribution; thus, we used nonparametric statistics that compare mean ranks rather than medians to compare differences between groups. Specifically, we used the Mann-Whitney *U* and Kruskal-Wallis *H* tests to examine whether the posted content differed by sex, ethnicity, and major. The Friedman test was used to determine whether the total number of tweets and percentage of tweets related to academic studies, personal health, and social life differed between nonexam (weeks 1-8) and final-exam (weeks 9 and 10) periods. To protect against type I error, we used the Bonferroni adjustment for post hoc comparison. Statistical significance was defined by a 2-tailed test with a *P* value <.05. All analyses were performed using IBM SPSS 20.0 (IBM Corporation).

## Results

### Participants

A total of 170 participants (women: 104, 61.2%) were included in our analysis. The mean age was 18.1, standard deviation (SD) 0.34 years. There was a wide range of distribution for ethnicity (white: n=33, 19.4%; African American: n=22, 12.9%; Latino: n=50, 29.4%; Asian: n=43, 25.3%; other minorities: n=27, 15.9%). The areas of study were health science (n=78, 45.9%), business (n=16, 9.4%), mathematics and engineering (n=21, 12.4%), social science and arts (n=30, 17.6%), and undeclared (n=30, 17.6%).

### Tweet Content During the Semester

**Figure 1:**
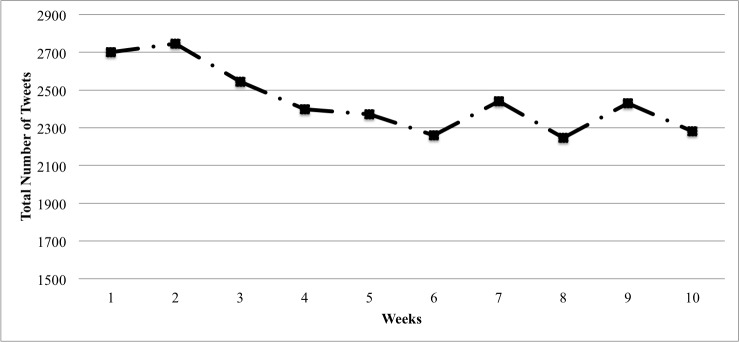
Total number of tweets throughout the semester (October 5 to December 13, 2015).

**Figure 2:**
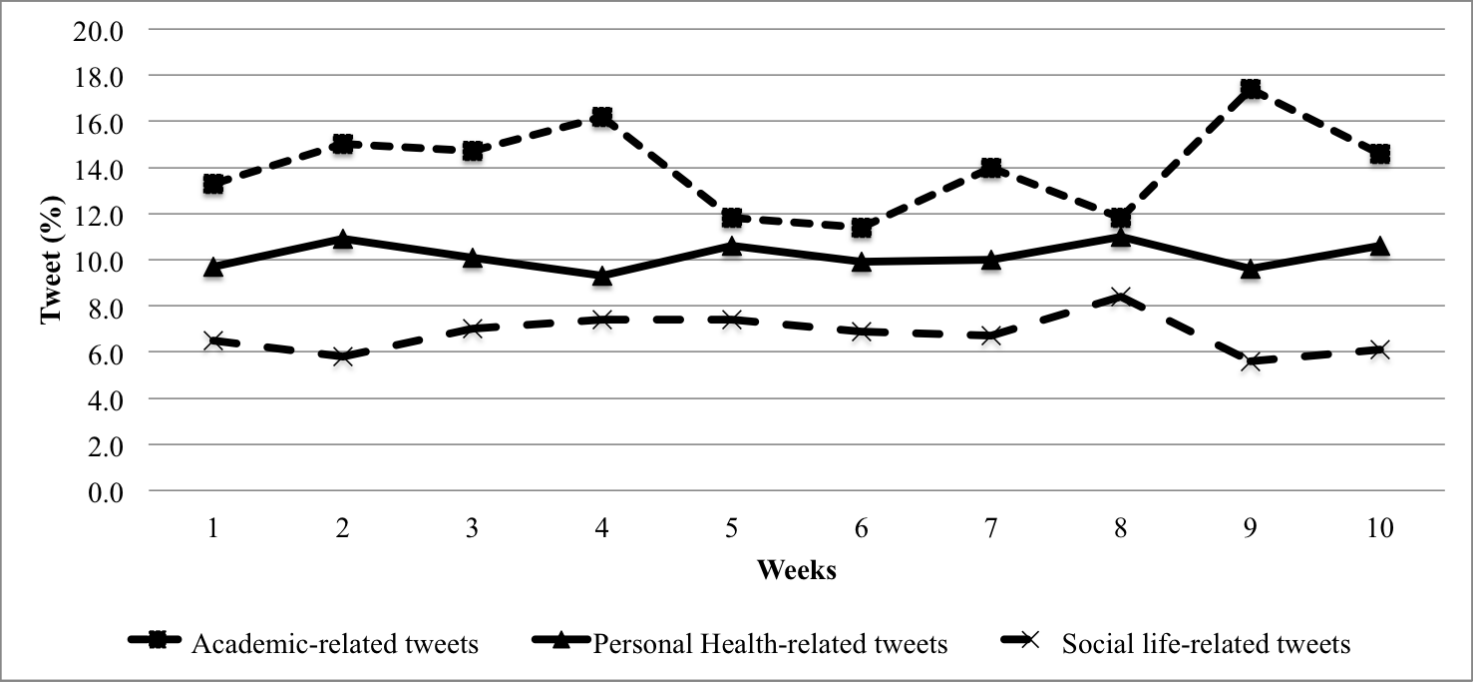
Proportion of total tweets related to academic studies, personal health, and social life during the semester (October 5 to December 13, 2015).

There were 24,421 tweets posted during the fall semester (October 5 to December 13; Figure 1). The mean number of tweets posted per participant throughout the fall semester was 138 (SD 210, range 3-1310). Table 1 displays the 10 most frequently used keywords related to academic study, personal health, and social life. Most of the participants tweeted about personal health, academic study, and social life. Specifically, we extracted tweets related to academic study, personal health, and social life-related tweets from 94.7% (n=161), 87.6% (n=149), and 94.1% (n=160) of the participants, respectively. Figure 2 shows the total percentage of weekly tweets related to academic study, personal health, and social life throughout the semester. Overall, academic-related tweets (n=3433, 14.06%) were the most prevalent during the entire semester, compared with tweets related to personal health (n=2483, 10.17%) and social life (n=1646, 6.74%). Since categories were not mutually exclusive, some tweets contained keywords from multiple categories. The overall overlap between all the categories was low: academic and social life overlap: 4.43% (n=223 tweets); academic and personal health overlap: 3.72% (n=446 tweets); social life and personal health overlap: 7.41% (n=308 tweets); and academic, social, and personal health overlap: 0.64% (n=49 tweets). We found that significantly fewer weekly tweets were posted during the final-exam period than the rest of the semester (final-exam weeks: mean rank 78.6, mean 13.3, standard error (SE) 1.5; nonfinal-exam weeks: mean rank 96.4, mean 14.0, SE 1.7; *Z*=–2.0, *P*=.04). The proportion of academic-related tweets increased during final-exam period compared with nonexam periods (final-exam weeks: mean rank 68.9, mean 18%, SE 0.1%; nonexam weeks: mean rank 80.7, mean 21%, SE 0.2%; *Z*=–2.1, *P*=.04). Meanwhile, the proportion of tweets related to social life decreased during final exams compared with nonexam periods (final-exam weeks: mean rank 70.2, mean 5.4%, SE 0.01%; nonfinal weeks: mean rank 81.8, mean 7.4%, SE 0.01%; *Z*=–4.8, *P*<.001). We found no significant difference for the proportion of personal health-related tweets between the final-exam and nonfinal-exam periods.

**Table 1:** The 10 most popular words in tweets associated with each category.

Rank	Academic study		Personal health and lifestyle		Social life	
	Word	Frequency	Word	Frequency	Word	Frequency
1	Class	634	Sleep	375	Friends	373
2	College	403	Running	163	Birthday	290
3	School	368	Stress	100	Music	156
4	Final	350	Sick	90	Weekend	153
5	Midterm	322	Hurt	70	Chill	132
6	Study	260	Walking	62	Party	113
7	Work	227	Gym	61	Friday	104
8	Quarter	225	Healthy	48	Concert	58
9	Essay	210	Workout	25	Drunk	54
10	Paper	182	Weights	14	Sex	24

**Table 2:** Weekly number of tweets posted during nonexam and exam periods by participants, by sex, ethnicity, and area of study.

Characteristics		Nonexam period (weeks 1-8)			Exam period (weeks 9 and 10)		
		Mean	SE^a^	Range	Mean	SE	Range
**Sex**							
	Female	14.7	11.9	0-90	14.9	2.1	0-98
	Male	12.8	13.0	0-148	10.8	2.2	0-96
**Ethnicity**							
	White	7.0	2.3	0-77	7.6	2.7	0-83
	African American	23.4	5.9	0-93	17.5	3.3	0-95
	Latino	19.0	14.0	0-148	17.3	4.2	0-84
	Asian	10.5	12.8	0-114	9.8	2.6	0-94
	Other minorities	12.3	12.8	0-48	15.8	5.3	0-98
**Area of study**							
	Health science	14.3	12.4	0-113	14.6	2.3	0-94
	Business	16.1	7.4	0-93	14.4	7.2	0-92
	Mathematics and engineering	9.1	2.1	1-33	8.3	1.9	0-31
	Social science and arts	19.0	5.3	0-148	16.8	4.0	0-98
	Undeclared	11.1	3.4	0-88	9.4	3.6	0-95

^a^SE: standard error.

### Sex and Tweet content

Women tweeted more often than men during both nonexam (women: mean rank 95.8; men: mean rank 76.8; *U*=2876, *P*=.02) and final-exam periods (women: mean rank 96.2; men mean rank 76.2; *U*=2832, *P*=.01; Table 2). However, during both nonexam and exam periods, the proportion of the tweets related to academic studies (nonexam: women’s mean rank 83.7, men’s mean rank 96.1, *U*=3157, *P*=.12; exam: women’s mean rank 77.4, men’s mean rank 84.7, *U*=2618, *P*=.34), personal health (nonexam: women’s mean rank 83.7, men’s mean rank 96.1, *U*=3157, *P*=.12; exam: women’s mean rank 75.8; men’s mean rank 87.7, *U*=2451, *P*=.11), and social life (nonexam: women’s mean rank 89.6, men’s mean rank 96.7, *U*=3549, *P*=.78; exam: women’s mean rank 80.1, men’s mean rank 80.0, *U*=2878, *P*=.98) were similar between the sexes (Table 3).

**Table 3:** Weekly percentage of tweets posted during nonexam and exam periods by participants, by sex, ethnicity, and area of study.

Characteristics		Academic tweets, mean % (SE^a^)		Personal health tweets, mean % (SE)		Social life tweets, mean % (SE)	
		Nonexam period	Exam period	Nonexam period	Exam period	Nonexam period	Exam period
**Sex**							
	Female	16.1 (1)	19 (1.9)	10.2 (1)	8.2 (0.9)	7.6 (0.6)	5.0 (1)
	Male	20 (2)	24 (3.3)	11 (0.9)	12.3 (2.2)	7.0 (0.7)	6.2 (2)
**Ethnicity**							
	White	19 (2.4)	32 (0.5)	10.4 (1.1)	6.7 (1.6)	8.9 (1.1)	7.4 (2.9)
	African American	16.5 (0.2)	12 (3)	10.1 (1.1)	6.9 (1.4)	8.2 (1.6)	5.9 (1.5)
	Latino	20 (2.4)	18.7 (2.1)	11.5 (1)	11.2 (2.0)	7.5 (1.0)	6.5 (1.8)
	Asian	18 (1.6)	23 (3.9)	9.2 (0.8)	11.4 (2.8)	5.3 (0.5)	2.5 (0.6)
	Other minorities	12 (2.0)	15.1 (4.5)	10.0 (2.6)	10.1 (2.7)	7.8 (1.1)	4.9 (1.9)
**Areas of study**							
	Health science	11.5 (1.0)	18.8 (1.7)	11.5 (1.0)	11.5 (1.9)	6.8 (1.1)	6.6 (1.4)
	Business	10.0 (2.1)	20.2 (0.4)	10.0 (2.1)	2.7 (1.2)	7.9 (1.1)	2.2 (1.2)
	Mathematics and engineering	9.3 (0.8)	21 (2.5)	9.3 (0.8)	7.4 (1.7)	7.9 (1.2)	4.9 (2.0)
	Social science and arts	8.1 (0.7)	13 (1.6)	8.0 (0.7)	5.7 (1.1)	6.9 (1.2)	6.5 (2.6)
	Undeclared	10 (1.4)	15.5 (2.3)	10.4 (1.4)	14.5 (2.6)	7.2 (1.0)	4.9 (2.0)

^a^SE: standard error.

### Ethnicity and Tweet Content

African Americans tweeted most frequently compared with other ethnic groups during both nonexam (χ^2^_4_=13.3, *P*=.01) and exam periods (χ^2^_4_=11.1, *P*=.03; Table 2). Interestingly, the percentages of academic-related tweets were similar between the ethnic groups during nonexam periods (χ^2^_4_=7.3, *P=*.12). However, during the final-exam periods, the percentage of academic tweets was significantly lower among African Americans than among whites (χ^2^_4_=15.1, *P*=.004). The proportion of academic-related tweets decreased only among African Americans and Latinos during the exam period compared with the nonexam period. During nonexam and exam periods, the percentages of tweets related to personal health (nonexam: χ^2^_4_=8.9, *P=*.08; exam: χ^2^_4_=3.8, *P*=.43) and social life (nonexam: χ^2^_4_=7.3, *P*=.11; exam: χ^2^_4_=6.9, *P*>.13) were not significantly different between ethnic groups (Table 2).

### Area of Study and Tweet Content

Students in social science and arts had the highest number of weekly tweets throughout the semester compared with participants in other majors. However, the mean difference was not significant (χ^2^_4_=3.5, *P=*.48). During nonexam and exam periods, the percentages of tweets related to academic studies (nonexam: χ^2^_4_=7.5, *P=*.11; exam: χ^2^_4_=9.1, *P*=.06), personal health (nonexam: χ^2^_4_=4.4, *P=*.35; exam: χ^2^_4_=4.6, *P*=.30), and social life (nonexam: χ^2^_4_=3.0, *P=*.55; exam: χ^2^_4_=5.2, *P=*.27) were not significantly different between areas of study (Table 3).

## Discussion

### Principal Findings

This study examined the content of the tweets of college freshman throughout a single semester. To our knowledge, this is the first study that examined the types of social media content posted and the frequency of content discussed among college freshmen. These results could have several important implications for academic researchers and school administrators.

First, our results suggest that the number of tweets related to academic studies and social life fluctuates to reflect real-time events. The proportion of academic tweets increased while the proportion of tweets related to social life decreased during the exam period compared with the nonexam period. This change in the proportion of tweets reflects the types of activities that students are experiencing at these different points in time and thus suggests that it may be possible to monitor students’ college experience through the study of their tweets. Previous studies have shown that the analysis of tweet sentiment (positive, negative, or neutral) can be used to monitor user experience [5-7]. A possible future application to monitoring student experience in real time is to combine the Twitter analytic methods used in this study with sentiment analysis. For example, tweets related to academic studies, personal health, or social life could be extracted. Sentiment analysis could then be applied to the categories to determine whether an individual’s attitude toward or perception of that category (eg, academic study or personal health) is positive, negative, or neutral. This study provides preliminary evidence to suggest that Twitter data can be used to monitor students’ experience related to academic study, personal health, social life. Future studies in this area are warranted.

Second, our results suggest that students’ ethnicity influenced the proportion of academic-related tweets they posted. During exam periods, African American students tweeted significantly less about academic studies than did white students, even though African American students tweeted more often in total than other ethnic groups. The difference in academic-related content may suggest academic disengagement [8]. Previous studies have shown that African Americans and Latinos have underperformed compared with white students due to factors such as differences in cultural patterns or socioeconomic status [9-11]. Social media data can offer an alternative method to study the influence of ethnicity and academic disengagement by providing another tool for understanding organic, real-time data on racial and ethnic differences. Furthermore, talking about academic studies on social media may be a way for students to share their academic experience and cope with academic-related stress. Previous studies have found that Latinos are more likely than whites to turn to family and friends for coping with academic-related stress [1,12]. Therefore, the difference in coping strategy (eg, family, friends, and online) between ethnic groups could be a contributing factor to differences in the academic-related tweets we observed. A potential future application for academic researchers and school administrators is to use social media to understand student behavior and engage with the students to promote academic studies.

Third, the overall number of weekly tweets of individual students related to academic studies, personal health, and social life remained relatively low, and the data were sparse. This means that a longer period of data collection would be required to reach a sufficient sample size to monitor students’ college experience on an individual level. The optimal amount of time required for data collection remains unclear. De Choudhury et al [13] conducted one of the first studies that used an individual’s tweets to predict risk of depression. The authors collected Twitter data over a 1-year period to build a model that was able to monitor and predict risk of depression in adults. Despite the small sample size and sparse data on an individual level observed in our study, Twitter data could be well suited to monitor students’ college experience on a population level. Study participants (n=170) posted between 100 and 300 weekly tweets related to academic study, personal health, and social life. A previous study found that the number of tweets related to risky sexual behavior and drug use was associated with HIV prevalence on a countywide level in the Untied states [14]. Similarly, tweets related to physical activity were negatively associated with obesity rates on a countywide level [15]. Academic researchers and school administrators could use similar methods to monitor students’ college experience by combining other data sources (eg, grade point average or stress level) with Twitter data. Future studies need to examine whether this method could offer real-time information on students’ college experience that school administrators and researchers could use to help improve this experience.

### Limitations

The study was not without limitations. We collected data during freshmen’s first semester at one university, and this was because this study was part of a larger study in which only freshman students were recruited. It is possible that the types and frequency of content expressed by students in other universities or upper years would be different. The students were aware that their tweets were being collected for research, which may have led to a bias in the form of self-censoring during the study period. Furthermore, 25% of Internet users are registered on Twitter [16]. It may be possible that these users differ from non-Twitter users in demographic characteristics, cultural background, or socioeconomic status. Nevertheless, the sample that we collected in this study included a wide range of ethnic groups and study majors. These factors need to be taken into consideration to ensure that Twitter content analyses are generalizable. Additionally, we were able to categorize up to 34% of all tweets into categories of academic studies, personal health, and social life by using keyword searches. It is possible that we missed certain tweets by excluding other keywords from our analyses. Future studies should also examine other topic modeling methods such as latent Dirichlet allocation.

### Conclusion

The ability to use social media data to provide real-time information on students’ postsecondary experience has significant implications for universities and school administrators. In this study, we found that it is feasible to extract tweets related to academic studies, personal health, and social life posted by college freshmen. The proportion of academic-related tweets increased, while tweets related to social life decreased during the exam period versus nonexam period. Finally, ethnicity influenced the types of tweet content. Specifically, during the exam period, African Americans tweeted less content related to academic studies than did whites. The findings from this study provide valuable information on the types of information that could be extracted from social media data posted by postsecondary students. Future studies need to examine whether school administrators and researchers may use this method combined with other Twitter analysis techniques (eg, sentiment analysis) to monitor students’ university experience in real time.

## Abbreviations

SD: standard deviation
SE: standard error
UCLA: University of California Los Angeles
